# Hormone‐related diseases and prostate cancer: An English national record linkage study

**DOI:** 10.1002/ijc.32808

**Published:** 2019-12-11

**Authors:** Eleanor L. Watts, Raphael Goldacre, Timothy J. Key, Naomi E. Allen, Ruth C. Travis, Aurora Perez‐Cornago

**Affiliations:** ^1^ Cancer Epidemiology Unit, Nuffield Department of Population Health University of Oxford Oxford United Kingdom; ^2^ Unit of Health‐Care Epidemiology, Big Data Institute NIHR Oxford Biomedical Research Centre, Nuffield Department of Population Health, University of Oxford Oxford United Kingdom; ^3^ Clinical Trial Service Unit and Epidemiological Studies Unit, Nuffield Department of Population Health University of Oxford Oxford United Kingdom

**Keywords:** epidemiology, IGF‐I, hormones, prostate cancer, record‐linkage, testosterone

## Abstract

Insulin‐like growth factor‐I (IGF‐I) and testosterone may be related to prostate cancer risk. Acromegaly is associated with clinically high IGF‐I concentrations. Klinefelter's syndrome, testicular hypofunction and hypopituitarism are associated with clinically low testosterone concentrations. We aimed to investigate whether diagnosis with these conditions was associated with subsequent prostate cancer diagnosis and mortality. We used linked English national Hospital Episode Statistics and mortality data from 1999 to 2017 to construct and follow‐up cohorts of men aged ≥35 years diagnosed with (*i*) acromegaly (*n* = 2,495) and (*ii*) hypogonadal‐associated diseases (*n* = 18,763): Klinefelter's syndrome (*n* = 1,992), testicular hypofunction (*n* = 8,086) and hypopituitarism (*n* = 10,331). We estimated adjusted hazard ratios (HRs) and confidence intervals (CIs) for prostate cancer diagnosis and death using Cox regression in comparison with an unexposed reference cohort of 4.3 million men, who were admitted to hospital for a range of minor surgeries and conditions (*n* observed cases = 130,000, *n* prostate cancer deaths = 30,000). For men diagnosed with acromegaly, HR for prostate cancer diagnosis was 1.33 (95% CI 1.09–1.63; *p* = 0.005; *n* observed cases = 96), HR for prostate cancer death was 1.44 (95% CI 0.92–2.26; *p* = 0.11; *n* deaths = 19). Diagnosis with Klinefelter's syndrome was associated with a lower prostate cancer risk (HR = 0.58, 95% CI 0.37–0.91; *p* = 0.02; *n* observed cases = 19) and hypopituitarism was associated with a reduction in prostate cancer death (HR = 0.53, 95% CI 0.35–0.79; *p* = 0.002; *n* deaths = 23). These results support the hypothesised roles of IGF‐I and testosterone in prostate cancer development and/or progression. These findings are important because they provide insight into prostate cancer aetiology.

## Abstract 2

**What's new?**

Although the incidence rates of prostate cancer vary globally, little is known about modifiable risk factors. Previous studies have shown insulin‐like growth factor‐I (IGF‐I) and testosterone may be related to prostate cancer risk. Using national hospital admission data, the authors examined whether these associations are consistent across clinical extremes in comparison with a reference cohort. Men diagnosed with acromegaly (characterised by high IGF‐I) had an increased risk of incident prostate cancer, while men diagnosed with diseases characterised by low testosterone had a lower risk of prostate cancer mortality. The findings support the role of IGF‐I and testosterone in prostate cancer pathogenesis.

**Abbreviations**
APC: admitted patient care
CI: confidence interval
HES: hospital episode statistics
HR: hazard ratio
ICD10: International Classification of Disease, 10th revision
IGF‐I: Insulin‐like growth factor‐I
KS: Klinefelter's syndrome
TH: testicular hypofunction
TRT: testosterone replacement therapy

## Introduction

Prostate cancer is the second most common cancer in men worldwide.^1^ Established risk factors include age, family history, ethnicity and genetic factors.^2^ Although there are large differences in global incidence rates, little is known regarding potentially modifiable risk factors. However, data from an international pooled individual‐level meta‐analysis of prospective studies have shown that high circulating insulin‐like growth factor‐I (IGF‐I) concentrations are associated with an increased risk of prostate cancer^3^ and that low free testosterone is associated with a reduced risk.^4^

Acromegaly is an endocrine disorder (often caused by a pituitary adenoma), characterised by hypersecretion of growth hormone by the pituitary gland, generally resulting in high circulating IGF‐I concentrations.^5^, ^6^, ^7^ Although acromegaly has previously been implicated with an increased overall cancer incidence,^8^ no study has had the statistical power to robustly investigate the association between acromegaly and prostate cancer incidence or mortality.

Klinefelter's syndrome (KS), testicular hypofunction (TH) and hypopituitarism are all conditions which are associated with clinically low circulating testosterone concentrations.^9^, ^10^, ^11^, ^12^, ^13^, ^14^ KS is a genetic abnormality caused by two or more X chromosomes in males and is the most common genetic cause of human male infertility.^14^, ^15^ TH and hypopituitarism are diagnoses used to describe a broad range of diseases, caused by both genetic mutations and/or pathophysiological processes.^10^, ^11^, ^12^, ^13^ TH is characterised by impaired testicular function and hypopituitarism by reduced anterior pituitary hormone secretion.^16^, ^17^ Observational studies have found some evidence that KS may be associated with a lower prostate cancer risk, but the evidence is inconclusive.^18^, ^19^, ^20^ Furthermore, it is not known whether this association is consistent across the other hypogonadal‐associated diseases that are characterised by low circulating testosterone concentrations.

Examining associations between these hormone‐related diseases and prostate cancer risk will help improve our understanding of the role of IGF‐I and testosterone in prostate cancer aetiology. Prospective cohort studies based on voluntary recruitment are generally underpowered to investigate rare diseases (such as acromegaly and KS); we have therefore used a linked dataset of routinely collected English national hospital episode statistics admitted patient care (HES APC) from 1999 to 2017 to test whether there was an association between (*i*) acromegaly and (*ii*) KS, TH and hypopituitarism with subsequent risk of prostate cancer diagnosis and mortality.

## Materials and Methods

### Study design and population

The methodology used was similar to that described previously.^21^, ^22^, ^23^ English national HES APC and mortality data from January 1, 1999 to March 31, 2017, were used to construct and follow‐up several cohorts of patients through record linkage. HES APC data comprises demographic and clinical data recorded for all patients undergoing admission to National Health Service (NHS) hospitals and treatment centres in England. HES APC covers both day case admissions (admission without an overnight stay) and inpatient care (at least one overnight stay). The records are linked so that individuals can be traced through multiple admissions over time.

The data resources were obtained for permitted use in our study and ethics approval was obtained from the Central and South Bristol Multi‐Centre Research Ethics Committee (04/Q2006/176) for analysis of the record‐linked data.

### Acromegaly and hypogonadal cohorts

The “exposure” cohorts were constructed to define the population of males diagnosed with acromegaly, KS, TH and hypopituitarism (Supporting Information Fig. S1). Cohorts of men were identified in the linked HES dataset using the International Classification of Disease, 10th revision [ICD‐10] codes E22.0 (acromegaly), Q98.0‐Q98.4 (KS), E29.1 (TH) and E23.0 (hypopituitarism). KS, TH and hypopituitarism are all characterised by low testosterone concentrations; therefore, these conditions were also combined into one cohort to maximise statistical power.

The inclusion criteria were men aged 35 years or more who had an admission during the study period with one of the exposure conditions, coded in any diagnosis position on the discharge record (i.e. either as a primary diagnosis or as a comorbidity during an admission for another medical or surgical procedure). Date of entry was based on the patient's earliest known date of hospital admission for the relevant condition. All participants who had a prior prostate cancer diagnosis, who had both acromegaly and hypogonadal‐associated diseases on their hospital records, or had missing covariate data (age, year of cohort entry, the region of residence, Index of Multiple Deprivation [IMD] rank; Supporting Information Fig. S1) were excluded.

It has been recommended that men who are treated for hypogonadal diseases with testosterone replacement therapy (TRT) are screened for prostate cancer,^24^ which may lead to detection bias. Therefore, we excluded all men who were diagnosed with prostate cancer within the first 6 months of cohort entry across all cohorts (Supporting Information Fig. S1).

### Reference cohort

A reference cohort of men with no known prior record of prostate cancer was constructed by identifying men aged 35 years or more without acromegaly, KS, TH or hypopituitarism (Supporting Information Fig. S1), who were admitted to hospital for various other conditions and injuries recorded as the primary diagnosis or main operation on the hospital record: strabismus, cataract, otitis, varicose veins, haemorrhoids, upper respiratory tract infections, nasal polyps, teeth disorders, inguinal hernia, nail diseases, sebaceous cyst, internal derangement of knee, bunions, vasectomy, dislocations/sprains/strains, bruising, gall bladder disease, appendicectomy, hip replacement, knee replacement or tonsillectomy. This diverse range of conditions was chosen on the basis that they are common and relatively minor, so that the men in the reference cohort would be broadly representative of the general population. We chose conditions/operations that were recorded as the primary diagnosis or operation in the hospital record to avoid selecting men who were coming into hospital principally for more serious or uncommon problems.

Date of entry to the reference cohort was based on the patient's earliest known date of admission for one of these conditions. As with the exposure cohorts, those who were diagnosed with prostate cancer within the first 6 months of cohort entry were excluded (Supporting Information Fig. S1).

### Follow‐up for prostate cancer diagnosis and vital status

From cohort entry, men were followed‐up through record linkage for any subsequent day‐case or inpatient admission for prostate cancer (ICD‐9 code 185, ICD‐10 C61), recorded either as a primary diagnosis or elsewhere on the hospital record. HES APC does not contain data on deaths occurring outside hospital. To censor for death and to identify further prostate cancer cases not otherwise recorded in hospital, we linked the HES APC records were linked to mortality records obtained from the Office for National Statistics, and assigned prostate cancer case if it appeared anywhere on the death record (Supporting Information Table S1). This register was also used to identify prostate cancer mortality. Tumour subtype data were not available in these datasets; therefore prostate cancer mortality was used as a marker of tumour aggressiveness. To capture these clinically aggressive tumours, prostate cancer death was defined as the underlying cause of death only (Supporting Information Table S1).

Our analysis was not linked to the cancer registry data. To estimate the extent of under‐ascertainment (i.e., the proportion of prostate cancer patients who were not identified using HES records or mortality data), the proportion of men with prostate cancer recorded in cancer registry data that did not have a concurrent prostate cancer HES APC and/or mortality record was calculated in the UK Biobank dataset. In this separate dataset HES APC and death data combined captured 76% of prostate cancer cases observed in the English cancer registry.

### Statistical analysis

Eligible men contributed person‐years from date of cohort entry until the date of first known prostate cancer diagnosis, death, or the end of the follow‐up period (March 31, 2017), whichever came first.

Multivariable Cox proportional hazards regression models were used to estimate hazard ratios (HRs) of (*i*) incident prostate cancer and (*ii*) prostate cancer mortality, comparing in turn each exposed cohort with the reference cohort. HRs were adjusted for age (in 5‐year groups), calendar year of first recorded admission (to remove period effects), region of residence (nine regions) and patients’ IMD (in fifths).^25^ Time from cohort entry was used as the underlying time variable. The analytical cohorts included 2,495 men with acromegaly, and 1,992, 8,086 and 10,331 men with KS, TH and hypopituitarism, respectively and 4.3 million in the reference cohort (Supporting Information Fig. S1).

Subgroup analyses were also conducted to assess whether associations differed by age at cohort entry (35–64; 65+ years) and by the length of follow‐up (6 months–4 years; 5+ years); these categories were chosen *a priori* due to possible differences in prostate tumours in younger men^26^ and detection bias.^24^ For these purposes, we fitted interaction terms between the subgroup variables and hormonal disorder diagnosis, and compared the models with and without the interaction terms using likelihood ratio tests to determine statistical significance.

All tests of statistical significance were two‐sided, and statistical significance was set at the 5% level. All statistical tests were undertaken using Stata/MP 14.0 (StataCorp, College Station, TX), and figures were created in R version 3.2.3.

## Results

Participant characteristics at cohort entry are displayed in Table 1. Most men entered the cohorts aged 35–64 years. Men in the KS cohort had higher levels of socioeconomic deprivation (Table 1). In the reference cohort, median follow‐up time was 7.7 years (interquartile range [IQR] 3.9–12.4), 127,299 men were diagnosed with prostate cancer and 29,022 died from the disease (as the underlying cause of death; Supporting Information Fig. S1).

**Table 1:** Participant characteristics at cohort entry

	Acromegaly (*n* = 2,495)	Klinefelter's Syndrome (*n* = 1,992)	Testicular hypofunction (*n* = 8,086)	Hypopituitarism (*n* = 10,331)	Reference cohort (*n* = 4,304,300)
Age group years, *n* (%)					
35–49	698 (28.0)	916 (46.0)	2,486 (30.7)	2,771 (26.8)	1,441,418 (33.5)
50–64	1,021 (40.9)	691 (34.7)	3,421 (42.3)	3,782 (36.6)	1,303,855 (30.3)
65–79	675 (27.1)	349 (17.5)	1,883 (23.3)	3,009 (29.1)	1,184,098 (27.5)
80+	101 (4.1)	36 (1.8)	296 (3.7)	769 (7.4)	374,929 (8.7)
Year of index admission, *n* (%)					
January 1999–2003	1,007 (40.4)	513 (25.8)	1,213 (15.0)	2,062 (20.0)	1,265,482 (29.4)
2004–2008	590 (23.7)	472 (23.7)	1,563 (19.3)	2,265 (21.9)	1,208,125 (28.1)
2009–2013	584 (23.4)	617 (31.0)	3,013 (37.3)	3,581 (34.7)	1,202,353 (27.9)
2014–March 2017	314 (12.6)	390 (19.6)	2,297 (28.4)	2,423 (23.5)	628,340 (14.6)
Government office region, *n* (%)					
North East	104 (4.2)	167 (8.4)	509 (6.3)	633 (6.1)	264,976 (6.2)
North West	305 (12.2)	326 (16.4)	1,709 (21.1)	1,774 (17.2)	643,095 (14.9)
Yorkshire and Humber	258 (10.3)	203 (10.2)	1,146 (14.2)	1,222 (11.8)	452,720 (10.5)
East Midlands	177 (7.1)	145 (7.3)	437 (5.4)	693 (6.7)	357,496 (8.3)
West Midlands	212 (8.5)	172 (8.6)	717 (8.9)	723 (7.0)	441,696 (10.3)
East of England	277 (11.1)	236 (11.9)	754 (9.3)	1,023 (9.9)	466,580 (10.8)
London	404 (16.2)	229 (11.5)	987 (12.2)	1,648 (16.0)	522,824 (12.2)
South East	464 (18.6)	294 (14.8)	1,131 (14.0)	1,533 (14.8)	658,130 (15.3)
South West	294 (11.8)	220 (11.0)	696 (8.6)	1,082 (10.5)	496,783 (11.5)
Index of multiple deprivation quintile, *n* (%)					
1 (highest deprivation)	463 (18.6)	523 (26.3)	1,831 (22.6)	2,112 (20.4)	796,636 (18.5)
2	461 (18.5)	451 (22.6)	1,632 (20.2)	2,042 (19.8)	832,001 (19.3)
3	535 (21.4)	395 (19.8)	1,567 (19.4)	2,080 (20.1)	897,729 (20.9)
4	519 (20.8)	371 (18.6)	1,624 (20.1)	2,094 (20.3)	916,192 (21.3)
5 (lowest deprivation)	517 (20.7)	252 (12.7)	1,432 (17.7)	2,003 (19.4)	861,742 (20.0)

Five cohorts in total are used in this analysis. All data excludes men diagnosed with prostate cancer within the first 6 months of follow‐up.

**Figure 1:**
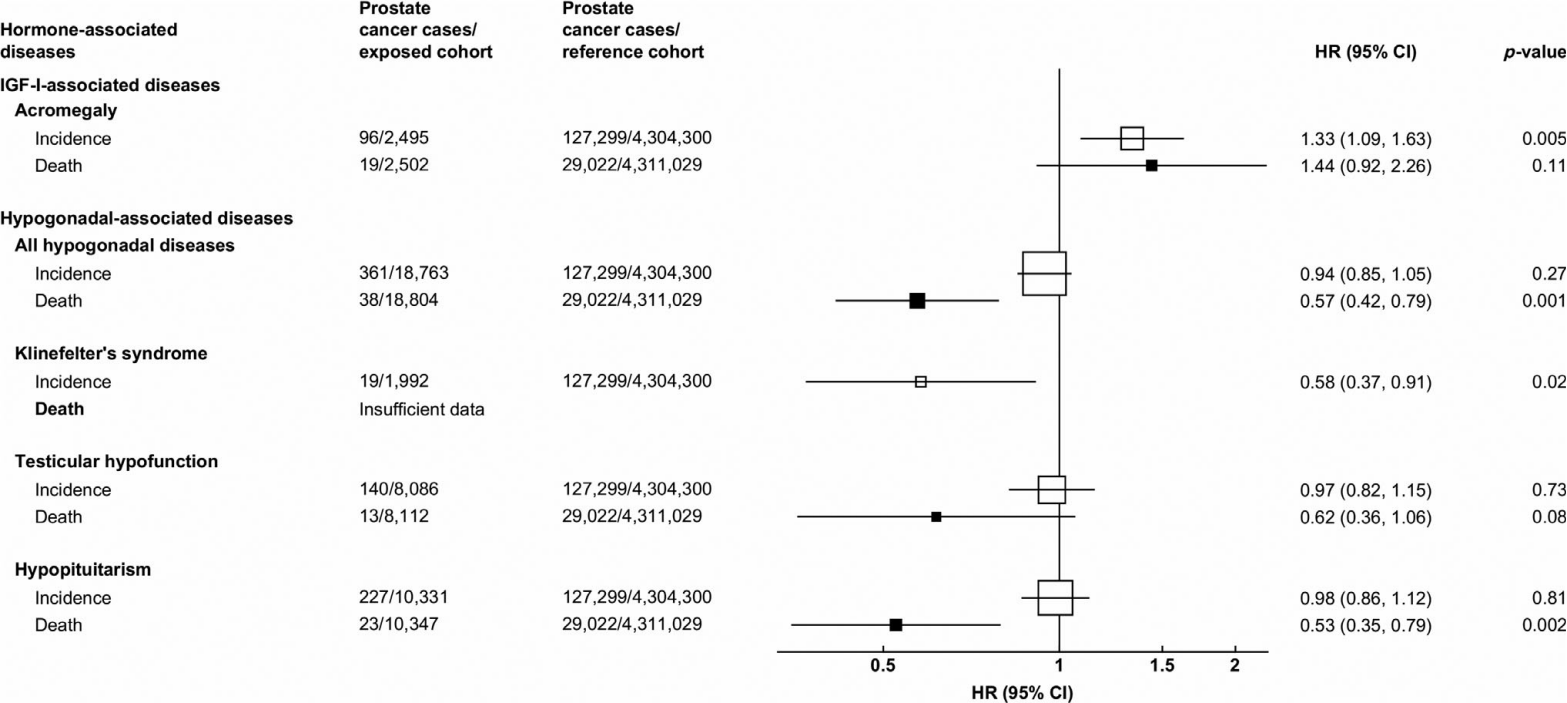
Hazard ratio* of prostate cancer incidence and mortality in men diagnosed with acromegaly, Klinefelter's syndrome, testicular hypofunction and hypopituitarism, in comparison with the reference cohort^†^. *HRs adjusted for age (5‐year groups), year of cohort entry, region of residence (nine regions), IMD rank (fifths). ^†^Conditions used in the reference cohort: strabismus, cataract, otitis, varicose veins, haemorrhoids, upper respiratory tract infections, nasal polyps, teeth disorders, inguinal hernia, nail diseases, sebaceous cyst, internal derangement of knee, bunions, vasectomy, dislocations/sprains/strains, bruising, gall bladder disease, appendectomy, hip replacement, knee replacement and tonsillectomy.

In the acromegaly cohort, median follow‐up time was 7.7 years (IQR 3.5–13.3), during which 96 cases of prostate cancer were diagnosed and 19 men died from prostate cancer. Compared to the reference cohort, acromegaly was associated with a 33% increased risk of being diagnosed with prostate cancer (HR = 1.33, 95% CI 1.09–1.63; *p* = 0.005), and a 44% increased risk of prostate cancer death (HR = 1.44, 95% CI 0.92–2.26; *p* = 0.11; Fig. 1).

In the hypogonadal cohorts, median follow‐up times were 6.8 (IQR 3.2–11.6), 4.9 (IQR 2.3–8.9) and 5.1 years (IQR 2.4–9.2) for the KS, TH and hypopituitarism cohorts, respectively. During these times 19, 140 and 227 men, respectively were diagnosed with prostate cancer, and 2, 13 and 23 prostate cancer deaths were recorded in each cohort, respectively. When all three hypogonadal cohorts were combined, there was no evidence of an association with risk of prostate cancer diagnosis (HR = 0.94; 95% CI 0.85–1.05; *p* = 0.27; based on 361 incident cases), but a significant reduction in prostate cancer death (HR = 0.57, 95% CI 0.42–0.79; *p* = 0.001; based on 38 deaths).

Compared to the reference cohort, men diagnosed with KS had a 42% lower risk of being diagnosed with prostate cancer (HR = 0.58, 95% CI 0.37–0.91; *p* = 0.02; Fig. 1). There was an insufficient number of prostate cancer deaths to investigate the association of KS with prostate cancer mortality. TH was not associated with overall risk of prostate cancer diagnosis (HR = 0.97, 95% CI 0.82–1.15; *p* = 0.73), but there was a suggestion of a reduction in prostate cancer mortality, based on 13 deaths (HR = 0.62, 95% CI 0.36–1.06; *p* = 0.08; Fig. 1). Similarly, hypopituitarism was not associated with overall prostate cancer diagnosis (HR = 0.98, 95% CI 0.86–1.12; *p* = 0.81), but was associated with a relative reduction in prostate cancer mortality, based on 23 deaths (HR = 0.53, 95% CI 0.35–0.79; *p* = 0.002; Fig. 1).

**Figure 2:**
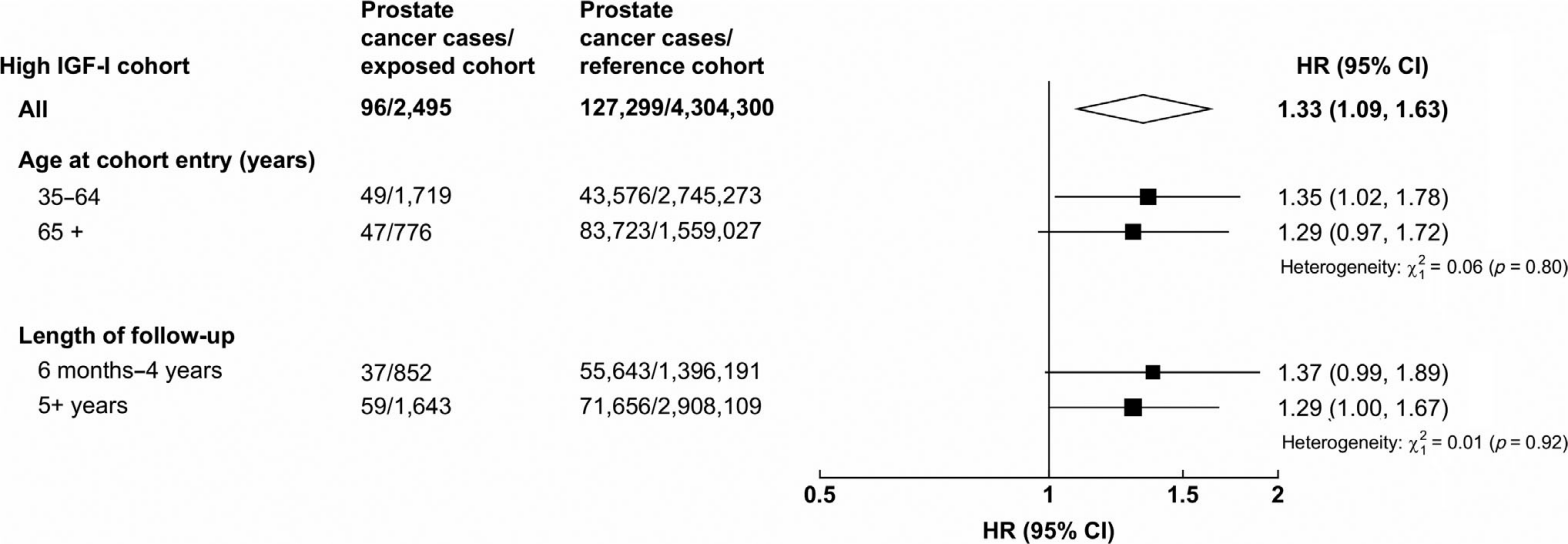
Hazard ratio* of prostate cancer incidence in men diagnosed with acromegaly in comparison with the reference cohort^†^, stratified by age at cohort entry and time interval. *HRs adjusted for age (5‐year groups), year of cohort entry, region of residence (nine regions), IMD rank (fifths). All figures exclude men diagnosed with prostate cancer within the first 6 months of follow‐up. ^†^Conditions used in the reference cohort: strabismus, cataract, otitis, varicose veins, haemorrhoids, upper respiratory tract infections, nasal polyps, teeth disorders, inguinal hernia, nail diseases, sebaceous cyst, internal derangement of knee, bunions, vasectomy, dislocations/sprains/strains, bruising, gall bladder disease, appendectomy, hip replacement, knee replacement and tonsillectomy.

**Figure 3:**
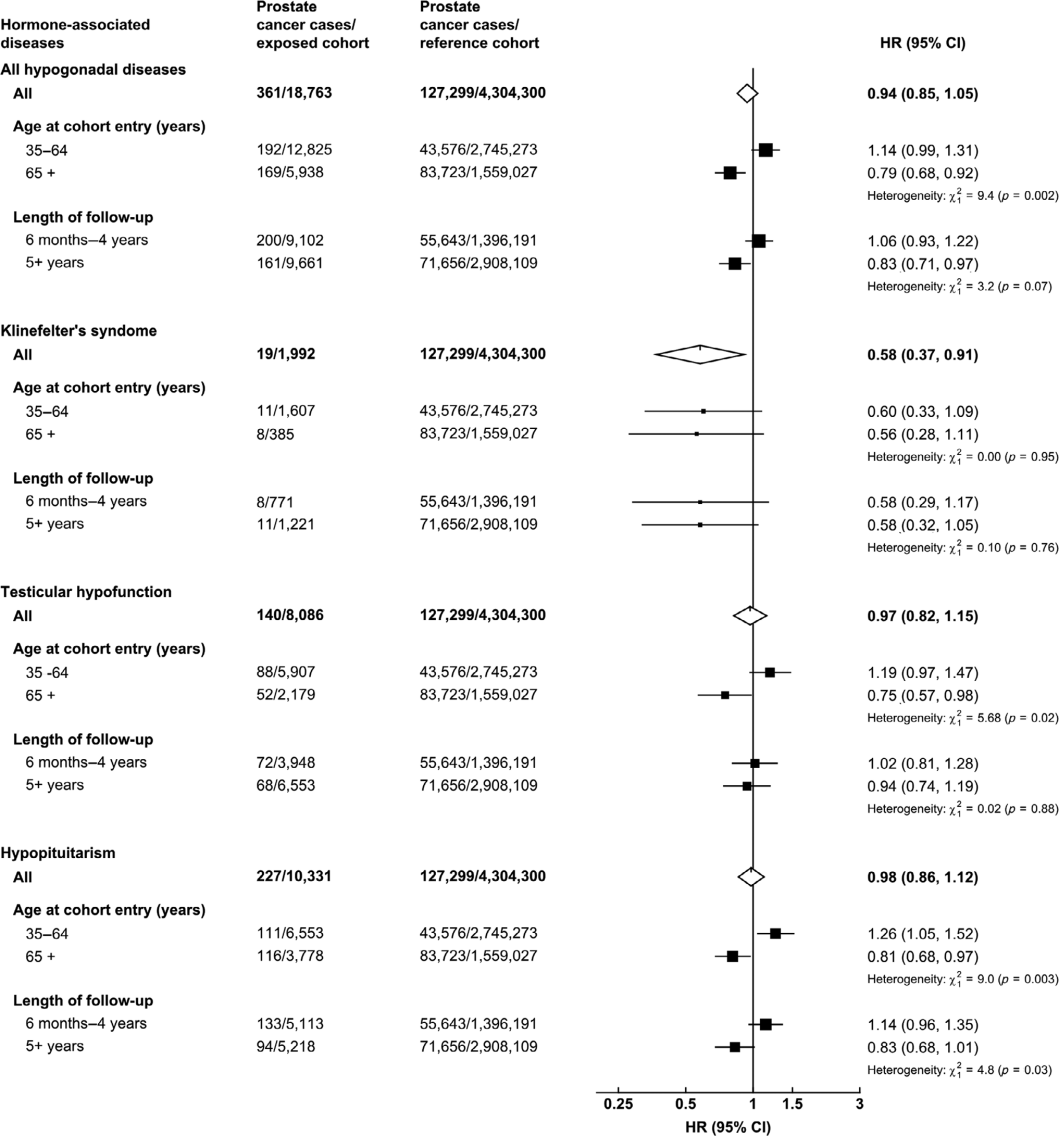
Hazard ratio* of prostate cancer incidence in men diagnosed with Klinefelter's syndrome, testicular hypofunction and hypopituitarism in comparison with the reference cohort^†^, stratified by age at cohort entry and time interval. *HRs adjusted for age (5‐year groups), year of cohort entry, region of residence (nine regions), IMD rank (fifths). All figures exclude men diagnosed with prostate cancer within the first 6 months of follow‐up. ^†^Conditions used in the reference cohort: strabismus, cataract, otitis, varicose veins, haemorrhoids, upper respiratory tract infections, nasal polyps, teeth disorders, inguinal hernia, nail diseases, sebaceous cyst, internal derangement of knee, bunions, vasectomy, dislocations/sprains/strains, bruising, gall bladder disease, appendectomy, hip replacement, knee replacement and tonsillectomy.

There was no evidence of heterogeneity in the associations of acromegaly or KS with prostate cancer diagnosis by age at cohort entry or length of follow‐up (Figs. 2 and 3). There was evidence of heterogeneity in the associations with incident prostate cancer by age at cohort entry in the TH and hypopituitarism cohorts. In both cohorts, only men aged 65+ years at cohort entry had a reduced risk of prostate cancer diagnosis in comparison with the reference cohort (Fig. 3). Men aged 35–65 years at cohort entry who were diagnosed with hypopituitarism had a significantly increased risk of being diagnosed with prostate cancer.

## Discussion

Our study of hormone‐related diseases and prostate cancer is the largest to date and the first to find statistically significant evidence of an association between diagnosis of acromegaly and an increased risk of incident prostate cancer. Diagnosis with any hypogonadal‐associated disease was associated with a reduced risk of prostate cancer mortality, and Klinefelter's syndrome was associated with a lower risk of prostate cancer diagnosis.

Acromegaly is associated with clinically high circulating IGF‐I concentrations,^5^, ^6^, ^7^ which may increase prostate cancer risk by increasing cell division and reducing programmed cell death.^27^ A recent meta‐analysis of 13 studies with data on acromegaly and prostate cancer reported a 20% increased risk of prostate cancer, however, the association was not statistically significant (based on 38 prostate cancer cases).^8^ Individual studies included were based on between 25 and 752 men with diagnosed acromegaly, and thus were not able to explore a possible association with prostate cancer mortality. Our study, based on 2,495 men diagnosed with acromegaly and 96 prostate cancer cases, found a 33% increased risk of prostate cancer diagnosis and a 44% increased risk of prostate cancer as the underlying cause of death. The former finding was statistically significant; the latter suggests a potentially strong association but was not statistically significant, perhaps owing to the limited number of prostate cancer deaths in this cohort (*n* = 19).

KS, TH and hypopituitarism are conditions associated with clinically low testosterone concentrations.^9^, ^10^, ^11^, ^12^, ^13^, ^14^ Low circulating testosterone concentrations may reduce prostatic androgen receptor signalling and may therefore reduce prostate cancer risk.^4^, ^28^, ^29^, ^30^, ^31^, ^32^ In our analysis, there was no association with prostate cancer diagnosis in the combined hypogonadal cohort, although men diagnosed with KS had a 42% lower risk. This is consistent with findings from other record linkage studies that have reported a 20–30% relative risk reduction of prostate cancer incidence in men with KS.^18^, ^19^, ^20^ To the best of our knowledge, this is the first study to specifically investigate TH or hypopituitarism and prostate cancer risk.

While men diagnosed with TH and hypopituitarism did not have a lower risk of prostate cancer incidence, they did have a lower risk of prostate cancer mortality (in the KS cohort the number of prostate cancer deaths was too low to investigate). Given that androgen deprivation therapy is a mainstay treatment for advanced prostate cancer,^33^ these results may indicate that a low androgen environment slows/inhibits prostate tumour growth, thereby reducing the risk of tumour progression and subsequent mortality, rather than affecting tumorigenesis.^34^

In this analysis, it is possible that there may be some sources of detection bias. TRT is a common treatment for hypogonadal diseases. Although there is no clear evidence that TRT promotes prostate tumorigenesis,^35^, ^36^ it is recommended that men receiving TRT are screened and regularly monitored for prostate cancer.^24^ Therefore, men receiving TRT may be more likely to attend prostate screening and so be diagnosed with prostate cancer. This screening recommendation may account for the lack of an observed inverse association with incident prostate cancer, and the heterogeneity in the associations by age observed in the TH and hypopituitarism cohorts (if screening is more common in younger men with these conditions). It may also partly account for the observed reduction in prostate cancer death in these men, if one assumes that screening leads to lower mortality. Although we excluded men diagnosed with prostate cancer within the first 6 months of cohort entry to reduce detection bias, we may not have eliminated this bias. The observation that men in the KS cohort but not in the TH or hypopituitarism cohorts had a lower risk of incident prostate cancer may be due to the life‐long exposure to a low testosterone environment in men with KS and/or or a lower prostate cancer screening attendance in these men, possibly related to their higher levels of socioeconomic deprivation.^37^, ^38^ There are currently no clinical guidelines recommending prostate cancer screening for men with acromegaly.^39^ A high proportion of prostate tumours will not become clinically relevant.^40^ By examining prostate cancer mortality as the underlying cause of death we were able to exclude asymptomatic or less aggressive tumours which are more likely to be detected due to bias.^41^

These analyses have some other limitations. Prostate tumour stage and grade were not available, although prostate cancer mortality was used as a proxy for clinical aggressiveness. We cannot exclude the possibility that other abnormalities which are associated with acromegaly and/or hypogonadal diseases may confound the associations.^20^ Although these diseases are informative proxy measures of aberrant hormone concentrations, no data were available regarding treatment, which aims to normalise hormone concentrations. Hence, it is unclear to what extent these men have abnormal hormone concentrations over the long‐term. This implies that the strengths of the associations found in our study may be underestimated. Further research could examine the possibility of shared genetic architecture between the exposure conditions and prostate cancer risk in order to further assess causality.

Although the investigated diseases were not required to be the principal diagnosis on the hospital record, we were only able to identify men who had hospital inpatient contact; therefore, we may not have identified all diagnoses of the exposure diseases or prostate cancer cases. Analysis of the separate UK Biobank dataset showed that approximately 24% of men with cancer registry data did not have a HES record of prostate cancer. Therefore, our cohort may represent men with more severe forms of both prostate cancer and the exposure diseases. Although this may be advantageous in the context of prostate cancer, this may bias our results if the extent of missing prostate cancer cases differs between the cohorts.

Strengths of this analysis include the large size of our study population and the person‐based cohort design using linked hospital records from an integrated healthcare system as well as mortality records. Coverage of all hospital records and all death records in England over a nearly 20‐year period enabled us to investigate associations across a variety of rare exposure diseases and prostate cancer diagnosis and mortality. We were also able to adjust for socioeconomic deprivation and explore interactions by follow‐up time. Our very large and heterogeneous reference cohort of 4.3 million men was designed to represent the general population in terms of prostate cancer risk while allowing comparability with the exposed cohorts for purposes of detailed analyses.

In summary, we found that men diagnosed with acromegaly have a higher risk of prostate cancer diagnosis and possibly prostate cancer mortality. Diagnosis of a hypogonadal‐associated disease was associated with a reduction in prostate cancer mortality, but there was only evidence of a relative reduction in prostate cancer diagnosis in the men who were diagnosed with KS. Overall, these results support the roles of IGF‐I and testosterone in prostate cancer development and/or progression. These associations provide insight into prostate cancer aetiology.

## Supporting information


**Figure S1** Schematic of cohort selection criteria
**Table S1**: Identification of prostate cancer by HES APC and death records

## Data Availability

The data that support the findings of our study are available from NHS Digital but restrictions apply to the availability of these data, which were used under licence for the current study, and so are not publicly available. Data are however available from the authors upon reasonable request and with permission of NHS Digital (https://digital.nhs.uk/).

## References

[1] Ferlay J , Ervik M , Lam F , et al. Global cancer observatory: cancer today. Lyon, France: International Agency for Research on Cancer, 2018.

[2] WCRF/AICR . World Cancer Research Fund/American Institute for Cancer research continuous update project: Diet, nutrition, physical activity, and prostate cancer. London: WCRF/AICR, 2014.

[3] Travis RC , Appleby PN , Martin RM , et al. A meta‐analysis of individual participant data reveals an association between circulating levels of IGF‐I and prostate cancer risk. Cancer Res 2016;76:2288–300. 26921328 10.1158/0008-5472.CAN-15-1551 PMC4873385

[4] Watts EL , Appleby PN , Perez‐Cornago A , et al. Low free testosterone and prostate cancer risk: a collaborative analysis of 20 prospective studies. Eur Urol 2018;74:585–94. 30077399 10.1016/j.eururo.2018.07.024 PMC6195673

[5] Lugo G , Pena L , Cordido F . Clinical manifestations and diagnosis of acromegaly. Int J Endocrinol 2012;2012:540398. 22518126 10.1155/2012/540398 PMC3296170

[6] Arosio M , Garrone S , Bruzzi P , et al. Diagnostic value of the acid‐labile subunit in acromegaly: evaluation in comparison with insulin‐like growth factor (IGF) I, and IGF‐binding protein‐1, ‐2, and ‐3. J Clin Endocrinol Metab 2001;86:1091–8. 11238491 10.1210/jcem.86.3.7288

[7] Melmed S . Acromegaly. N Engl J Med 2006;355:2558–73. 17167139 10.1056/NEJMra062453

[8] Dal J , Leisner MZ , Hermansen K , et al. Cancer incidence in patients with acromegaly: a cohort study and meta‐analysis of the literature. J Clin Endocrinol Metab 2018;103:2182–8. 29590449 10.1210/jc.2017-02457

[9] Smyth CM , Bremner WJ . Klinefelter syndrome. Arch Intern Med 1998;158:1309–14. 9645824 10.1001/archinte.158.12.1309

[10] Regal M , Páramo C , Sierra JM , et al. Prevalence and incidence of hypopituitarism in an adult Caucasian population in northwestern Spain. Clin Endocrinol (Oxf) 2001;55:735–40. 11895214 10.1046/j.1365-2265.2001.01406.x

[11] Comtois R , Beauregard H , Somma M , et al. The clinical and endocrine outcome to trans‐sphenoidal microsurgery of nonsecreting pituitary adenomas. Cancer 1991;68:860–6. 1855185 10.1002/1097-0142(19910815)68:4<860::aid-cncr2820680431>3.0.co;2-4

[12] Paja M , Lucas T , Garcia‐Uria J , et al. Hypothalamic‐pituitary dysfunction in patients with craniopharyngioma. Clin Endocrinol (Oxf) 1995;42:467–73. 7621564 10.1111/j.1365-2265.1995.tb02664.x

[13] Seftel A . Male hypogonadism. Part II: Etiology, pathophysiology, and diagnosis. Int J Impot Res 2005;18:223. 10.1038/sj.ijir.3901365 16094414

[14] Lanfranco F , Kamischke A , Zitzmann M , et al. Klinefelter's syndrome. Lancet 2004;364:273–83. 15262106 10.1016/S0140-6736(04)16678-6

[15] Morris JK , Alberman E , Scott C , et al. Is the prevalence of Klinefelter syndrome increasing? Eur J Hum Genet 2007;16:163–70. 18000523 10.1038/sj.ejhg.5201956

[16] Ascoli P , Cavagnini F . Hypopituitarism. Pituitary 2006;9:335–42. 17077946 10.1007/s11102-006-0416-5

[17] van Aken MO , Lamberts SW . Diagnosis and treatment of hypopituitarism: an update. Pituitary 2005;8:183–91. 16508719 10.1007/s11102-006-6039-z

[18] Hasle H , Mellemgaard A , Nielsen J , et al. Cancer incidence in men with Klinefelter syndrome. Br J Cancer 1995;71:416–20. 7841064 10.1038/bjc.1995.85 PMC2033611

[19] Swerdlow AJ , Schoemaker MJ , Higgins CD , et al. Cancer incidence and mortality in men with Klinefelter syndrome: a cohort study. J Natl Cancer Inst 2005;97:1204–10. 16106025 10.1093/jnci/dji240

[20] Jianguang J , Bengt Z , Jan S , et al. Risk of solid tumors and hematological malignancy in persons with turner and Klinefelter syndromes: a national cohort study. Int J Cancer 2016;139:754–8. 27061708 10.1002/ijc.30126

[21] Keenan TDL , Goldacre R , Goldacre MJ . Associations between obstructive sleep apnoea, primary open angle glaucoma and age‐related macular degeneration: record linkage study. Br J Ophthalmol 2017;101:155–9. 27044342 10.1136/bjophthalmol-2015-308278

[22] Pakpoor J , Goldacre R , Schmierer K , et al. Testicular hypofunction and multiple sclerosis risk: a record‐linkage study. Ann Neurol 2014;76:625–8. 25131454 10.1002/ana.24250

[23] De Pablo‐Fernandez E , Goldacre R , Pakpoor J , et al. Association between diabetes and subsequent Parkinson disease: a record‐linkage cohort study. Neurology 2018;91:e139–e42. 29898968 10.1212/WNL.0000000000005771

[24] Rhoden EL , Morgentaler A . Risks of testosterone‐replacement therapy and recommendations for monitoring. N Engl J Med 2004;350:482–92. 14749457 10.1056/NEJMra022251

[25] Department for Communities and Local Government . The English index of multiple deprivation (IMD) 2015‐guidance. London, UK: Department for Communities and Local Government, 2015.

[26] Salinas CA , Tsodikov A , Ishak‐Howard M , et al. Prostate cancer in young men: an important clinical entity. Nat Rev Urol 2014;11:317–23. 24818853 10.1038/nrurol.2014.91 PMC4191828

[27] Fürstenberger G , Senn H‐J . Insulin‐like growth factors and cancer. Lancet Oncol 2002;3:298–302. 12067807 10.1016/s1470-2045(02)00731-3

[28] DiGiovanni J , Kiguchi K , Frijhoff A , et al. Deregulated expression of insulin‐like growth factor 1 in prostate epithelium leads to neoplasia in transgenic mice. Proc Natl Acad Sci USA 2000;97:3455–60. 10737798 10.1073/pnas.97.7.3455 PMC16261

[29] Thompson IMJ , Goodman PJ , Tangen CM , et al. Long‐term survival of participants in the prostate cancer prevention trial. N Engl J Med 2013;369:603–10. 23944298 10.1056/NEJMoa1215932 PMC4141537

[30] Andriole GL , Bostwick DG , Brawley OW , et al. Effect of dutasteride on the risk of prostate cancer. N Engl J Med 2010;362:1192–202. 20357281 10.1056/NEJMoa0908127

[31] Thompson IM , Goodman PJ , Tangen CM , et al. The influence of finasteride on the development of prostate cancer. N Engl J Med 2003;349:215–24. 12824459 10.1056/NEJMoa030660

[32] Heinlein CA , Chang C . Androgen receptor in prostate cancer. Endocr Rev 2004;25:276–308. 15082523 10.1210/er.2002-0032

[33] Mottet N , Bellmunt J , Bolla M , et al. EAU‐ESTRO‐SIOG guidelines on prostate cancer. Part 1: screening, diagnosis, and local treatment with curative intent. Eur Urol 2017;71:618–29. 27568654 10.1016/j.eururo.2016.08.003

[34] Huggins C , Stevens RE Jr , Hodges CV . Studies on prostate cancer: II. The effects of castration on advanced carcinoma of the prostate gland. Arch Surg 1941;43:209–23.

[35] Rhoden Ernani L , Morgentaler A . Testosterone replacement therapy in hypogonadal men at high risk for prostate cancer: results of 1 year of treatment in men with prostatic intraepithelial neoplasia. J Urol 2003;170:2348–51. 14634413 10.1097/01.ju.0000091104.71869.8e

[36] Snyder PJ , Ensrud KE , Lewis CE , et al. Lessons from the testosterone trials. Endocr Rev 2018;39:369–86. 29522088 10.1210/er.2017-00234 PMC6287281

[37] Littlejohns TJ , Travis RC , Key TJ , et al. Lifestyle factors and prostate‐specific antigen (PSA) testing in UKbiobank: implications for epidemiological research. Cancer Epidemiol 2016;45:40–6. 27693812 10.1016/j.canep.2016.09.010 PMC5147810

[38] Cremante A , Clerici F , Destefani V , et al. Klinefelter's syndrome and psychoneurologic function. Mol Hum Reprod 2010;16:425–33. 20197378 10.1093/molehr/gaq018

[39] Katznelson L , Laws ER Jr , Melmed S , et al. Acromegaly: an Endocrine Society clinical practice guideline. J Clin Endocrinol Metab 2014;99:3933–51. 25356808 10.1210/jc.2014-2700

[40] Draisma G , Boer R , Otto SJ , et al. Lead times and overdetection due to prostate‐specific antigen screening: estimates from the European randomized study of screening for prostate cancer. J Natl Cancer Inst 2003;95:868–78. 12813170 10.1093/jnci/95.12.868

[41] Jahn JL , Giovannucci EL , Stampfer MJ . The high prevalence of undiagnosed prostate cancer at autopsy: implications for epidemiology and treatment of prostate cancer in the prostate‐specific antigen‐era. Int J Cancer 2015;137:2795–802. 25557753 10.1002/ijc.29408 PMC4485977

