# Supplementary material for: Hormone‐related diseases and prostate cancer: An English national record linkage study
Source: Int J Cancer. 2019 Dec 11;147(3):803–10. doi: 10.1002/ijc.32808 (PMC7318262; doi:10.1002/ijc.32808)
Supplement: Supplementary file 1 — Figure S1 Schematic of cohort selection criteria Table S1: Identification of prostate cancer by HES APC and death records [file IJC-147-803-s001.pdf]

Hormone-related diseases and prostate cancer: an English national record linkage study: Supplementary Materials

Authors

Eleanor L. Watts, Raphael Goldacre, Timothy J. Key, Naomi E. Allen, Ruth C. Travis,

Aurora Perez-Cornago.

Table of contents

Figure S1: Schematic of cohort selection criteria………………...…………………….....Page 2

Supplementary Table S1: Identification of prostate cancer by HES APC and death records…………………….………………………………………………..Page 3

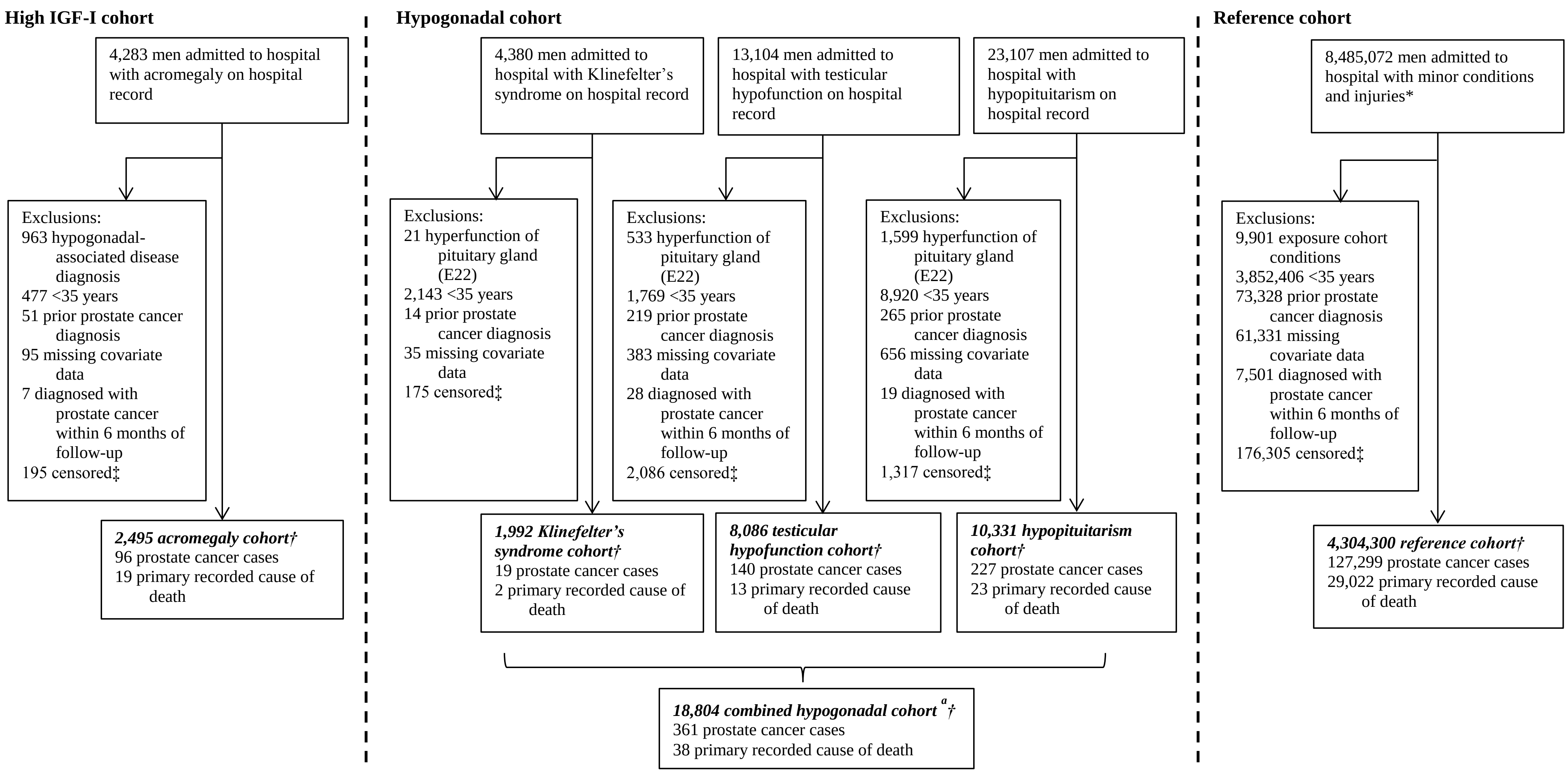

Supplementary Figure S1: Schematic of cohort selection criteria

* Conditions used in the reference cohort: strabismus, cataract, otitis, varicose veins, hemorrhoids, upper respiratory tract infections, nasal polyps, teeth disorders, inguinal hernia, nail diseases, sebaceous cyst, internal derangement of knee, bunions, vasectomy, dislocations/sprains/strains, bruising, gall bladder disease, appendectomy, hip replacement, knee replacement, tonsillectomy.

† Cohort numbers refers to the men included in the prostate cancer incident analysis. For the analysis with prostate cancer mortality men who were diagnosed with prostate cancer within the first 6 months of follow-up, but did not die within this time were included in this analysis. These men were excluded in the analysis of incident prostate cancer.

‡ Censoring due to men who died within the first 6 months of follow-up, or those who reached 31st March 2017 within the first 6 months follow-up.

[a] Hypogonadal disease cohorts are not mutually exclusive therefore the combined hypogonadal cohort may sum to less than the individual hypogonadal disease cohorts.

Supplementary Table S1: Identification of prostate cancer by HES APC and death records

Endpoint		High IGF-I cohort	Hypogonadal cohort			Reference cohort
		**Acromegaly**	**Klinefelter's syndrome**	**Testicular hypofunction**	**Hypopituitarism**	**Reference conditions†**
Prostate cancer cases	Total	96	19	140	227	127,334
	Death record only*	4 (4.2%)	0 (0.0%)	1 (0.7%)	8 (3.5%)	5,737 (4.5%)
Prostate cancer deaths	Primary cause of death only	19	2	13	23	29,022

*Anywhere on death record.
† Conditions used in the reference cohort: strabismus, cataract, otitis, varicose veins, hemorrhoids, upper respiratory tract infections, nasal polyps, teeth disorders, inguinal hernia, nail diseases, sebaceous cyst, internal derangement of knee, bunions, vasectomy, dislocations/sprains/strains, bruising, gall bladder disease, appendectomy, hip replacement, knee replacement, tonsillectomy.
% value denotes the percentage of the total cases that were identified from death record only (i.e. no hospital record of prostate cancer).
Abbreviations: HES APC=Hospital Episode Statistics Admitted Patient Care; IGF-I=insulin-like growth factor-I
